# Combined computed tomography and fluorodeoxyglucose positron emission tomography in the diagnosis of prosthetic valve endocarditis: a case series

**DOI:** 10.1186/1756-0500-7-32

**Published:** 2014-01-13

**Authors:** Michele Bartoletti, Fabio Tumietto, Giovanni Fasulo, Maddalena Giannella, Francesco Cristini, Rachele Bonfiglioli, Luigi Raumer, Cristina Nanni, Silvia Sanfilippo, Marco Di Eusanio, Pier Giorgio Scotton, Maddalena Graziosi, Claudio Rapezzi, Stefano Fanti, Pierluigi Viale

**Affiliations:** 1 Infectious Diseases Unit, Department of Medical and Surgical Sciences, Policlinico S. Orsola Malpighi, Alma Mater Studiorum Università di Bologna, Bologna, Italy; 2 Nuclear Medicine, Infectious Diseases Unit, Department of Medical and Surgical Sciences, Policlinico S. Orsola Malpighi, Alma Mater Studiorum Università di Bologna, Bologna, Italy; 3 Department of Cardiovascular Surgery, Sant’Orsola-Malpighi Hospital, University of Bologna, Bologna, Italy; 4 Infectious Disease Unit, Treviso City Hospital, Treviso, Italy; 5 Cardiology, Azienda Ospedaliero-Universitaria di Bologna, Policlinico S. Orsola-Malpighi, Bologna, Italy

**Keywords:** FDG-PET, Prosthetic valve endocarditis, Diagnosis, Duke’s criteria

## Abstract

**Background:**

The diagnosis of prosthetic valve endocarditis is challenging. The gold standard for prosthetic valve endocarditis diagnosis is trans-esophageal echocardiography. However, trans-esophageal echocardiography may result in negative findings or yield images difficult to differentiate from thrombus in patients with prosthetic valve endocarditis. Combined computed tomography and fluorodeoxyglucose positron emission tomography is a potentially promising diagnostic tool for several infectious conditions and it has also been employed in patients with prosthetic valve endocarditis but data are still scant.

**Case presentations:**

We reviewed the charts of 6 patients with prosthetic aortic valves evaluated for suspicion of prosthetic valve endocarditis, at two different hospital, over a 3-year period. We found 3 patients with early-onset PVE cases and blood cultures yielding *Pseudomonas aeruginosa, Staphylococcus epidermidis* and *Staphylococcus lugdunensis*, respectively; and 3 late-onset cases in the remaining 3 patients with isolation in the blood of *Streptococcus bovis, Candida albicans* and *P. aeruginosa,* respectively. Initial trans-esophageal echocardiography was negative in all the patients, while fluorodeoxyglucose positron emission tomography showed images suspicious for prosthetic valve endocarditis. In 4 out of 6 patients valve replacement was done with histology confirming the prosthetic valve endocarditis diagnosis. After an adequate course of antibiotic therapy fluorodeoxyglucose positron emission tomography showed resolution of prosthetic valve endocarditis in all the patients.

**Conclusion:**

Our experience confirms the potential role of fluoroseoxyglucose positron emission tomography in the diagnosis and follow-up of prosthetic valve endocarditis.

## Background

The ageing population with prolonged life expectancy and the improvement of surgical procedures determines that a greater number of patients may live with prosthetic valve and/or implantable cardiac devices [1,2]. Prosthetic valve endocarditis (PVE) remains a severe complication of valve surgery, still carrying a high mortality and accounting for 1% to 6% of valve replacement [3,4]. Due to the high prevalence of comorbidities in patients with PVE and the growing incidence of difficult-to-treat pathogens in healthcare-associated infection, [5] an early diagnosis and an aggressive therapeutic management is mandatory [1].

The gold standard for diagnosis of PVE is trans-oesophageal echocardiography (TEE); however TEE can be non conclusive or falsely negative in the initial phases of the disease and it should be repeated if initially negative [6]. Moreover in patients with cardiac devices, the usual imaging of endocarditis at TEE is less frequent.

Positron emission tomography (PET) and computed tomography (CT), are routinely used for the diagnosis and follow up of malignancies, based on the increased glycolytic rate in malignant cells. Similarly, many infective and inflammatory conditions can be imaged with PET due to the increased uptake of [7] F-Fluorodesossiglucose by inflammatory cells (granulocytes, monocytes as well as lymphocytes) and granulation tissue as these cells use glucose as an energy source [8,9]. In fact, combined computed tomography and fluorodeoxyglucose positron emission tomography (CT/FDG-PET) is increasingly used for the management of several infectious conditions, mainly when associated to vascular devices: it was successfully used in patients with prosthetic vascular graft infections, allowing the diagnosis with a sensitivity (SE) of 88-93% and specificity (SP) of 70-91% [10,11]. In addition, SE and SP of FDG-PET alone for aortic graft infection were 91% and 64%, compared with SE and SP of CT that were 64% and 86%, thus a higher benefit with combined CT/FDG-PET is expected [12]. Moreover, CT/FDG-PET may be an excellent tool for the diagnosis of bacterial embolism and metastatic infections difficult to detect, such as mycotic aneurysms [13-15]. Finally, the CT/FDG-PET allows to calculate the standardized uptake value (SUV), a reproducible quantitative parameter useful for assessing the efficacy of therapy in the follow-up [16].

Despite this promising rationale, the current literature on the role of CT/FDG-PET in the diagnosis of PVE is scant and mostly based on case reports. Here we report a case series of PVEs with initial negative echocardiography in whom the PVE diagnosis was achieved using CT/FDG-PET.

## Case presentations

### Case 1

A 42-year-old male patient with a history of previous intravenous drug abuse and osteosynthesis implant on the left femur in 2004, was admitted to our hospital in February 2010 for persistent fever. One month before admission, he underwent major heart surgery with graft replacement of aortic valve, aortic root and ascending aorta (Bentall procedure). On admission, his physical examination was unremarkable with exception of fever. Chest X-ray was negative, blood tests showed white-blood cells (WBC) count of 10.420 cells/mm^3^ and C-reactive protein 17.13 mg/dL. The patient underwent transthoracic echocardiography (TTE) followed by TEE and signs of infection were excluded. He was treated with clindamycin and meropenem for 3 weeks with resolution of fever.

**Figure 1:**
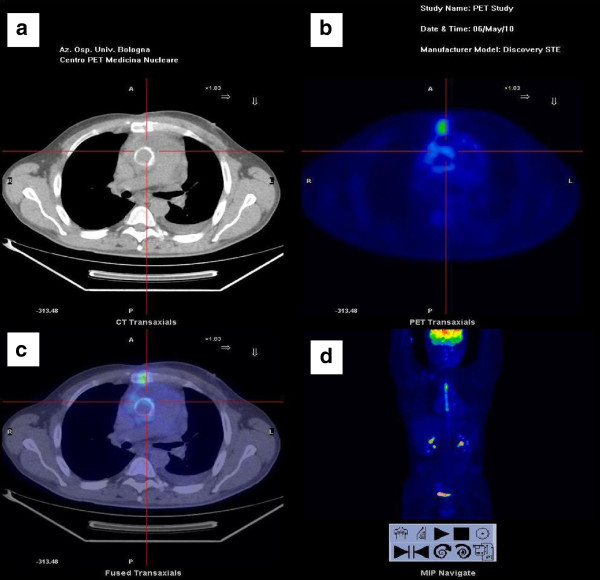
**Case 1: the image below shows a focal uptake (SUVmax = 3.5) at anterior wall of mechanical aortic prostethic valve, consistent with an acute infection. a**. CT. **b**. PET. **c**. Fusion image. **d**. Maximum intensity projection.

A few days after antibiotic discontinuation, fever relapsed and he was transferred to our Infectious Disease Unit. A repeated TEE was once again negative; blood cultures yielded a *Pseudomonas aeruginosa* strain susceptible to all tested antibiotics*.* Because of the high clinical suspicion of PVE, a CT/FDG-PET was performed, and revealed a focal uptake, of aortic valve and ascending aorta consistent with infection with maximal standardized uptake value (SUVmax) of 3.5 (Figure 1). The patient underwent surgical replacement of the Bentall prosthesis and was treated with levofloxacin for 4 weeks. Intraoperative and histological results confirmed the PVE diagnosis. At 1-year follow-up the patient showed no further evidence of PVE by TTE/TEE and CT/FDG-PET.

### Case 2

A 53-year-old male was admitted in July 2012 to our unit for a sepsis with isolation of a fluconazole-susceptible *Candida albicans* from blood cultures. He had a history of aortic valve replacement with mechanical prosthesis in May 2010. He underwent abdominal surgery for intestinal volvulus in April 2012 complicated with secondary peritonitis and candidemia by a fluconazole-susceptible *C. albicans* treated with a 3-week course of fluconazole.

At admission, TEE excluded cardiac involvement and treatment with fluconazole was started again. Despite adequate therapy and clinical response, blood cultures (+14 days) still yielded a fluconazole susceptible *C. albicans*. TEE was repeated one week after admission being still negative for valve vegetation or other signs of cardiac involvement. Despite the negative TEE, the patient developed a popliteal artery embolism that was managed with a Fogarty-catheter thromboembolectomy. A CT/FDG-PET was done showing a focal uptake (SUVmax = 3.2) on the mechanic aortic valve. Combination antifungal therapy with liposomal amphotericin B plus caspofungin was started and the patient underwent emergency mechanical aortic valve replacement. Intra-operatory findings and histological examination were consistent with PVE. The physical exploration and the laboratory findings at 6-month follow-up are unremarkable, both TTE/TEE and CT/FDG-PET tested negative as well.

### Case 3

A 71-year-old was admitted on September 2012 to a medical ward for persistence of fever despite an empirical antibiotic course. His medical history revealed a composite replacement of the aortic root and valve with Bentall procedure for an ascending aortic aneurysm with aortic insufficiency in October 2010.

On admission, there were no signs of vegetation and no abscess formation at TTE. After antibiotics discontinuation, blood cultures yielded *Streptococcus bovis* and a treatment with ceftriaxone was initiated with resolution of fever. One week later TEE revealed good prosthetic valve function without vegetation or other signs of PVE. However, due to the high suspicion of endocarditis a CT/FDG-PET was performed at the end of the second week of treatment showing uptake on aortic valve graft (SUVmax = 3.8) and upper pole of spleen (SUVmax = 7.4). Three weeks later, despite the appropriate antimicrobial treatment, the patient developed clinical signs of cardiac failure, thus a new TEE was performed revealing a small peri-prosthetic mass (7 mm) suggestive for an abscess formation. This feature was confirmed by a new CT/FDG-PET demonstrating higher focal uptake (SUVmax = 4.2). The patient underwent a surgical valve and Bentall prosthesis replacement. Intraoperative findings and histological examination confirmed PVE diagnosis. At 1 year follow-up the patient is symptom-free and in good clinical condition, no further evidence of an infectious process has been found by TTE/TEE and CT/FDG-PET.

### Case 4

A 46-year-old man was admitted on November 2011 to our hospital for remittent fever. He had a medical history of severe aortic stenosis underwent mechanical prosthesis replacement fifteen years before and re-operated in 2010 for valve dysfunction with a Bentall procedure. A month before admission he had been successfully treated as outpatient with a short antibiotic course with levofloxacin for a community-acquired pneumonia.

At admission, blood cultures yielded ceftazidime and piperacillin resistant *Pseudomonas aeruginosa*; TEE was negative and CT of aorta did not show leak, defects or peri-prosthetic collections. The patient was started on a 4 week-regimen with meropenem and ciprofloxacin, with favourable clinical course and discharge home. One month later, he was re-admitted for fever relapse. A *P. aeruginosa* strain with the same susceptibility pattern of the prior isolate was again isolated from the blood cultures. TEE was unremarkable, but CT/FDG-PET showed high uptake on lateral and anterior prosthetic wall (SUV = 10) and peri-valve tissue (SUV = 6).

Surgical treatment was deemed unfeasible for the high patient EUROscore (European System for Cardiac Risk Evaluation). Antimicrobial treatment with meropenem and ciprofloxacin was maintained for 4 weeks and then switched to suppressive life-long therapy with ciprofloxacin. The patient remained symptom-free and a CT/FDG-PET performed one year later showed a 50% reduction of focal uptake.

### Case 5

A 59-year-old man was admitted to our hospital for an ileocolic artery thrombosis and fever in January 2012. His past medical records revealed an aortic biological prosthestic valve replacement for severe stenosis in October 2011, complicated in the post-operative period by a bloodstream infection due to methicillin-resistant *Staphylococcus epidermidis* with negative TEE.

On admission TEE revealed a good valve function and no sign of vegetation. However, due to the persistence of low grade fever a CT/FDG-PET was performed showing a high uptake on the annulus of the aortic prosthetic valve (SUVmax = 10). He was started on daptomycin, oxacillin and rifampin and surgical valve replacement was scheduled. A preoperative TEE showed a 12.7 mm valve vegetation as well as surgical and histological findings confirmed the diagnosis of infective endocarditis. After a total 6 weeks of antibiotic therapy the patient was discharged, at 6-month follow-up the patient is free from disease, as confirmed by a new TTE and CT/FDG-PET.

### Case 6

A 80-year-old woman was admitted for fever, hypotension and confusion in March 2012. She had a history of aortic valve replacement with biological prosthesis and double coronary artery by-pass in April 2011.

At admission, blood cultures yielded a *Staphylococcus lugdunensis*. The patient was started on oxacillin with resolution of fever. The diagnostic work-up, including TEE and CT-angiogram of the aorta, was negative for PVE; whereas CT/FDG-PET revealed a diffuse uptake on prosthetic valve (SUVmax 4.2). Two weeks after hospital admission a new TEE showed a peri-valve tissue abscess with aortic vegetation. Surgical treatment was considered at high risk, thus the patient continued on antimicrobial treatment without recurrence of fever and improvement of general conditions. After 6 week of treatment a new CT/FDG-PET confirmed a reduction of the focal uptake. The patient was discharged on long-term antibiotic therapy, at 6-month follow-up she is free from symptoms.

## Discussion

We described six patients with aortic prosthetic valve and high clinical suspicion of PVE. In all of them the TEE was firstly negative, and only in two the TEE became further positive. In all cases CT/FDG-PET showed a high uptake on the peri-prosthetic valve level. The diagnosis of PVE was then confirmed by histology in all the 4 patients who underwent surgery. In the remaining 2 patients in whom the surgery was deemed as unfeasible, a reduction of the uptake on the prosthetic valve was documented by follow-up with CT/FDG-PET under long-term antibiotic therapy (Table 1).

**Table 1:** Prosthetic valve endocarditis cases diagnosed by combined computed tomography and fluorodeoxyglucose positron emission tomography

**Case N°**	**Prosthetic valve**	**Blood cultures**	**Initial TEE exploration**	**Repeated TEE findings**	**Surgical intervention**	**Histological confirmation**
**1**	Bentall composite graft	*Pseudomonas aeruginosa*	Negative	Negative	Y	Y
**2**	Mechanic aortic valve	*Candida albicans*	Negative	Negative	Y	Y
**3**	Bentall composite graft	*Sreptococcus bovis*	Negative	Small peri-prosthetic abscess	Y	Y
**4**	Bentall composite graft	*Pseudomonas aeruginosa*	Negative	Negative	N	_
**5**	Biological aortic valve	MRSE	Negative	12.7 mm vegetation	Y	Y
**6**	Biological aortic valve	*Staphylococcus lugdunensis*	Negative	Peri-valve abscess	N	_

*MRSE*, Methicillin resistant *Staphylococcus epidermidis*; *TEE*, transesophageal echocardiography; *Y*, Yes; *N*, No.

The major Duke’s criteria include positive blood cultures and evidence of cardiac involvement at TTE/TEE both for native and prosthetic endocarditis. However this indication, targeting TEE as the gold standard for instrumental diagnosis, could be disappointing in PVE. In fact, its performance may be sub-optimal for detecting the classical features of endocarditis such as vegetation because the damage due to biofilm-producing microorganisms may be localized at the level of valve annulus or peri-valve tissue resulting in leakage [17,18]. In addition, a highly skilled well-trained operator and patient compliance are needed for an optimal performance of the echocardiography tool.

Conversely, CT/FDG-PET owing its ability to demonstrate the metabolic activity of organs and tissues may allow earlier diagnosis, as in our cases. Another advantage of CT/FDG-PET is the possibility to quantify the standardized uptake value (SUV), this can be useful to assess the amount and extension of local infection. Additionally, CT/FDG-PET shows concomitantly the presence of secondary infection foci such as embolisms and/or metastatic infections. This tool is of pivotal value either for establishing the timing of surgery in those patients with brain embolisms, either for deciding the duration of antimicrobial therapy [7]. Indeed, the quantification of SUV may provide early information on the long term response to antibiotic therapy in patients not selected for surgery [16]. In fact, as we have already demonstrated in the setting of vertebral osteomyelitis, a correct antibiotic exposure quickly reduces the metabolic activity of the infectious process. Indeed, the change in SUV at two weeks of antibiotic treatment provides important information on the outcome, and it is predictive of the long term response [16]. In the current series, 2 patients had not received surgery, in both of them a repeat CT/PET during appropriate antibiotic course [19] showed reduction or disappearance of focal uptake. On the other hand, the high influence of antibiotic treatment on SUV (the longer is the treatment, the biggest the decline) represents the Achilles’ heel of the CT/FDG-PET diagnostic yield. Thus, the first SUV quantification should be assessed prior to any antibiotic treatment. In fact in our series, 3 out of 6 cases showed a low initial SUV, ranging from 3.2 to 3.8, very likely because of they were largely treated with antibiotics before carrying out the CT/FDG-PET. Despite this limitation, diagnosis of PVE was successfully achieved in all 6 patients, enabling a prompt surgical intervention in 4 of them. Therefore, a routine application of CT/FDG-PET, performed as soon as possible, without previous antibiotic exposure could improve significantly the diagnostic yield and clinical usefulness of CT/FDG-PET.

Although literature data is mainly based on case reports or small case series, like ours, the possible role of nuclear medicine tools in the diagnosis of infective endocarditis continues to be hypothesized [20]. Thus, the time for an update of Duke criteria specifically regarding the PVE, could be come: in PVE, CT/FDG-PET could be a true alternative/complementary imaging tool, when TTE/TEE is negative or inconclusive (for instance in patients with poor acoustic window) [14].

## Conclusions

In conclusion, CT/FDG-PET is a novel useful tool for early diagnosis of occult infectious processes, including PVE. The most consistent hypothesis is that this method is able to offer some diagnostic elements (such as a metabolic active process) earlier than TEE, allowing earlier diagnosis. However, its actual sensitivity and specificity for native and prosthetic valve endocarditis, as well as for other infections (e.g. cardiac implantable electronic devices), still have to be validated in larger prospective studies.

## Consent

Written informed consents were obtained from all of the patients in this case series for publication of this Case Report and any accompanying images. Copies of the written consents are available for review by the Editor-in-Chief of this journal.

## Abbreviations

PVE: Prosthetic valve endocarditis; TEE: Trans-esophageal echocardiography; CT: Computed tomography; FDG-PET: Fluorodeoxyglucose positron emission tomography; CT/FDG-PET: Combined computed tomography and fluorodeoxyglucose positron emission tomography; SE: Sensitivity; SP: Specificity; SUV: Standardized uptake value; TTE: Trans-thoracic echocardiography.

## Competing interests

The authors declare that they have no competing interests.

## Authors’ contribution

MB, FT, RB, CN, PGS, FC, PV, SF, MG LR and GF have made substantial contributions to conception and acquisition of data; MB, MG, FT, PV, PGS, CR, RB and CN have been involved in drafting the manuscript and revising it critically. All authors gave final approval for publication.
